# Design of Cross-Platform Information Retrieval System of Library Based on Digital Twins

**DOI:** 10.1155/2022/7999091

**Published:** 2022-09-27

**Authors:** Shanshan Shang, Zikai Yu, Kun Jiao, Yingshi Huang, Hua Guo, Guozhong Wang

**Affiliations:** ^1^Library, Shanghai University of Engineering Science, Shanghai 201620, China; ^2^Assembly Department, Shanghai Aerospace Equipments Manufacturer, Co., Ltd., Shanghai 200245, China; ^3^Institute of Artificial Intelligence Industry, Shanghai University of Engineering Science, Shanghai 201620, China

## Abstract

In order to improve the library's ability of cross-platform information retrieval and data scheduling and distribution, a library cross-platform information retrieval system based on digital twin technology is designed. Using data warehouse decision support and data source structured query methods, the spectral characteristics of Library cross-platform information resources are extracted. Using the method of Hadoop data parallel loading, the library cross-platform operation data is divided into decision-making data, computing resource pool data, and Hadoop parallel loading data. A library cross-platform information digital twin parallel retrieval and information fusion feature matching model is established, and the retrieval channels are allocated through multiple complex and balanced task scheduling sequences. According to the queue configuration model of Library cross-platform information retrieval, the optimization design of Library cross-platform information retrieval system is realized. The simulation test results show that the designed system has good recall ability of cross-platform information retrieval data, and improves the utilization rate of cross-platform resources and the dynamic scheduling ability of online resources.

## 1. Introduction

The design of Library cross-platform information retrieval system is based on the analysis of cloud data quality of service (QoS) [1], combined with the load parameter analysis of mesos slave node. The method of dynamic scheduling of cloud resources is used to extract the characteristics of cross-platform virtual resource allocation of the library [2]. Using virtual machine matching and dynamic node adaptive allocation methods, the design of Library cross-platform information retrieval system is realized.

The design of library cross-platform information retrieval system is based on the analysis of the Quality of Service (QoS) of cloud data [3], combined with the load parameter analysis of Mesos-Slave nodes, using the method of dynamic scheduling of cloud resources, extracting the characteristics of library cross-platform virtual resource allocation, and using the methods of virtual machine matching and dynamic node adaptive allocation to realize the design of library cross-platform information retrieval system [4]. The design methods of library cross-platform information retrieval system mainly include QoS dynamic resource node scheduling method, CaaS (Container-as-a-Service) scheduling method, and particle swarm optimization scheduling method. In 2011, the National Institute of Standards and Technology, NIST) proposes to use Tanimoto coefficient as the characteristic quantity of stable matching between container and virtual machine to carry out cross-platform information retrieval and resource scheduling in libraries, but the adaptability level of this method for cross-platform scheduling in libraries is not high. Reference [5] designed a library information retrieval system based on big data analysis technology. Firstly, the functions of library information retrieval system are described, and the overall framework of library information retrieval system is established; then the hardware subsystem and software subsystem of library information retrieval are designed in detail, and the library information retrieval algorithm is described in detail. However, this method has a large computational cost, and the reliability allocation ability of physical machine selection is poor [6, 7].

In view of the above problems, this paper proposes a library cross-platform information retrieval system based on digital twin technology. According to the extracted spectrum features of Library cross-platform information resources, a digital twin parallel retrieval and information fusion feature matching model is established. Through the dynamic allocation of multiple complex and balanced task scheduling sequences, the optimization design of Library cross-platform information retrieval model is realized. The experimental results show that this method has better advantages in improving the cross-platform information retrieval ability of the library.

## 2. Overall Structure Design and Functional Components of the System

### 2.1. Overall Structure Design of the Cross-Platform Information Retrieval System of the Library

**Figure 1:**
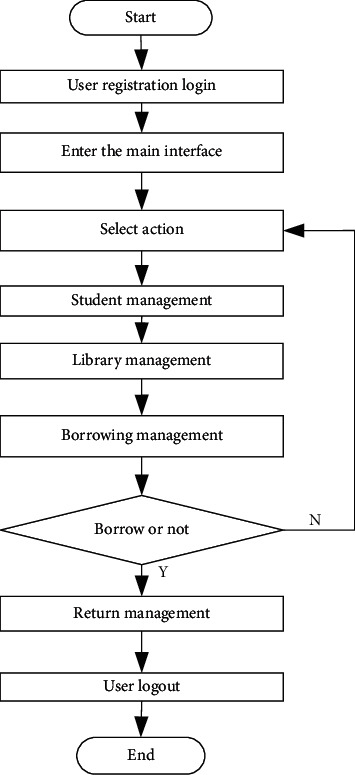
Library cross-platform information retrieval flow chart.

In order to realize the design of cross-platform information retrieval system of library based on digital twin technology, combining with the middleware design scheme, the API Server is established, the Kubernetes node model is stored, combining with the analysis of Web API (API server is an important management API layer of k8s). It is responsible for providing restful API access endpoints and persisting data to etcd server. In the kubernetes cluster, the API server acts as the location of the interaction portal. API server is not only responsible for interacting with etcd (other components will not directly operate etcd, only API server does so) but also provides a unified API call entry. All interactions are centered on API server. Storage kubernetes node model (kubernetes, or k8s for short) is an abbreviation that replaces the eight characters “ubernet” in the middle of the name with eight. It is an open source application used to manage containerized applications on multiple hosts in the cloud platform. Kubernetes' goal is to make the deployment of containerized applications simple and efficient. Kubernetes provides a mechanism for application deployment, planning, updating, and maintenance. The traditional application deployment method is to install applications through plug-ins or scripts. The disadvantage of this is that the operation, configuration, management, and all life cycles of the application will be bound to the current operating system. This is not conducive to the upgrade, update/rollback of the application. Of course, some functions can be realized by creating virtual machines. However, virtual machines are very heavy and not conducive to portability. The new method is implemented by deploying containers. Each container is isolated from each other. Each container has its own file system. Processes between containers will not affect each other, and computing resources can be distinguished. Compared with virtual machines, containers can be deployed quickly. Because containers are decoupled from underlying facilities and machine file systems, they can be migrated between different clouds and different versions of operating systems. Containers occupy less resources and deploy faster. Each application can be packaged into a container image. The one-to-one relationship between each application and the container also gives the container greater advantages. Containers can be used to create container images for applications at the build or release stage, because each application does not need to be combined with other application stacks, nor does it depend on the production environment infrastructure, which enables a consistent environment from R & D to testing and production. Similarly, containers are lighter and more “transparent” than virtual machines, which are easier to monitor and manage, and Web API (Message services are conceptually similar to traditional middleware). Due to the technical and commercial complexity, they have not been developed on a large scale. The web-based communication service visible in the short term is Amazon Simple Queue Service. This service facilitates secure and scalable queue based communication between any application. There is no general web computing service black box that can be accessed through API, but there are many technologies pointing in this direction. The first is ALexa vertical search platform, which will be mentioned more in the search service section below. The second is grid computing, such as sun grid, datasynapse's gridserver, or platform's symphony. Encapsulating arbitrary computing tasks in the API is a very challenging task, and it may take many years for this service to become widely popular. Information services provide a large amount of specific information, including geographic data like Google Maps API, product data like Amazon e-commerce, Amazon historical pricing services. and the latest Yahoo! Answer's API, etc. What these services have in common is that they all provide simple APIs to access massive amounts of data, which may lead to unpredictable cross applications between isolated information. Because of the foundation and dominance of search in the web domain, search services constitute a key part of the new web infrastructure. Google Search API is an earlier and now a typical search abstraction mechanism. Another example is the Alexa search platform, whose design has led to a series of vertical search engines that challenge Google's position. It is quite interesting that technically, the Alexa search platform is more like a computing service, but it is limited to the search field. This means the possibility of other services, such as sorting services or data transformation services. The last category I broadly call Web2.0 services. The name is not necessarily relevant, but it includes services such as del.icio.us, flickr, basecamp. John Musser compiled some very influential APIs in programmable web. These specific services will become the users of other services mentioned above in the future, but their value is more reflected in the fact that they provide clear, specific, and simple APIs to view and change the information we have. Although they look more like molecules than atoms, they are such basic services in today's web domain that it makes sense to treat them as components. New web platforms are changing the rules of the game. With the leverage of these infrastructures, it is possible to launch complex and intelligent applications in a very compact time slice. The mere fact that developers do not have to worry about scaling the problem is encouraging. In other words, Amazon's ten-year experience in large-scale distributed computing was immediately presented to everyone at a very feasible price. It is possible to build intelligent web applications or desktop applications that make full use of the power of these web services because these applications do not have to worry about infrastructure, but focus more on availability, ease of use, context and semantics! Storage API. In order to unify and standardize the operation APIs of these clients, the storage API is introduced. Through the storage API, we can view the available storage space and the used space, and even control whether the user needs to be reminded when the user data are cleared. Harbo functional components. In the early versions, the functions of harbor mainly focused on the management of docker images. Harbor developers hope that users can push and pull images simultaneously through a unified address, and use the graphical interface to browse and manage images. As for the push and pull functions, docker's open source distribution project is widely used. It can support different types of storage, and is relatively mature and stable. Therefore, harbor chooses distribution to handle the push and pull requests for client images, and provides management functions by adding other components around distribution. On the one hand, this method reduces the development workload; on the other hand, since distribution is basically the de facto standard of the image warehouse, it ensures the stability of the image push and pull functions. Later, with the iteration of the version, harbor gradually reduced its dependence on distribution. However, in terms of image read-write, access, and other functions, distribution is still a bridge between harbor and user storage. The random link node forwarding control is adopted to realize the design of ES log service, monitoring service, alarm service, and other functional modules of library cross-platform information retrieval system [8–12]. The specific process is shown in Figure 1.

**Figure 2:**
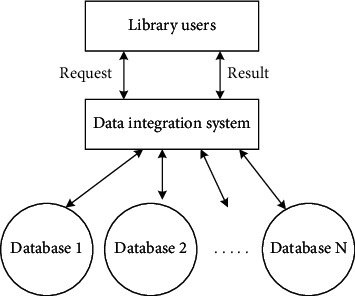
Data integration system model of cross-platform information retrieval system in library.

Data sharing is the key technology of library cross-platform information retrieval, which means that users in different places, using different computers and different software, can read the data stored in other systems, and can perform various operations, calculations, and analyses on library cross-platform information. If you want to share cross-platform information in libraries, you should first integrate cross-platform information and data in libraries. You need to analyze the characteristics of heterogeneous data sources, formulate a series of standards and specifications to realize the standardized design of cross-platform information retrieval system in libraries, and process data to achieve the purpose of integration. On this basis, you should provide access interfaces to users. Users do not need to care about the specific sources of data but only need to get the data they want through the provided data access interface for cross-platform information retrieval in libraries [13]. The data integration system model of library cross-platform information retrieval system is shown in Figure 2.

**Figure 3:**
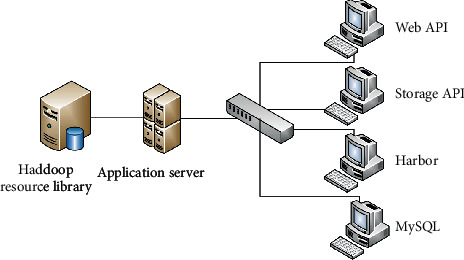
System logic framework of cross-platform information retrieval in libraries.

Library cross-platform information metadata is descriptive information of data and information resources, that is, data describing data. The metadata of HDFS is composed of the attributes, affiliation, and distribution location of Block. In HDFS, the management and maintenance of library cross-platform information metadata is completed by NameNode, which is the single failure point of the whole file system [14]. To ensure the reliability of cross-platform information metadata in libraries, HDFS uses two persistent ways, editlog and fsimage, to store metadata in disk. Among them, editlog records the historical information of cross-platform information metadata operation in the library in the form of operation log, and saves it after the record is completed; the fsimage is a kind of stored image file, which is mainly aimed at the checkpoint of regular backup of library cross-platform information metadata in HDFS steps and are expressed as follows: In the application resource pool, on the basis of the resource pool conversion of Slave node, a new log file is generated by the notification of Secondary NameNode, which is deployed in the access and retrieval node, and the data are fed into MySQL. The system logic framework of cross-platform information retrieval in the library is shown in Figure 3.

**Figure 4:**
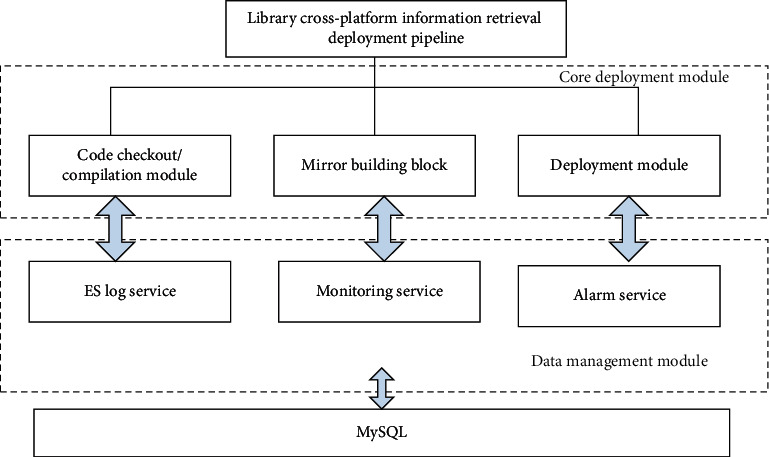
Architecture diagram of cross-platform information retrieval system in library.

According to the system logic framework of library cross-platform information retrieval shown in Figure 2, based on image service, authentication service, and ES log service control methods, the node automatic deployment system model of library cross-platform information retrieval is established [15]. The mirror image of compilation environment is pulled from Harbor warehouse, and Jenkins Master static scanning method is adopted to establish the distribution model of library cross-platform information retrieval system assembly line and core integrated deployment module [16]. The data extraction and program compilation of library cross-platform information retrieval are realized in MySQL underlying cloud database, and the dynamic monitoring model of library cross-platform information retrieval is established from the perspectives of source code construction, code compilation, and image construction, and the architecture diagram of library cross-platform information retrieval system is obtained, as shown in Figure 4.

According to the library cross-platform information retrieval system shown in Figure 3, the data warehouse decision support and data source structured query method are adopted to extract the spectrum characteristic quantity of library cross-platform information resources, and Dockerfile is used to generate the mirror image. The cross-platform information data packet of application library is deployed to Kubernetes cluster, and the functions of creating, configuring, executing, and deleting the cross-platform of library are realized through the module management of application package [17].

### 2.2. Library Cross-Platform Information Retrieval Module Function Component Analysis

**Figure 5:**
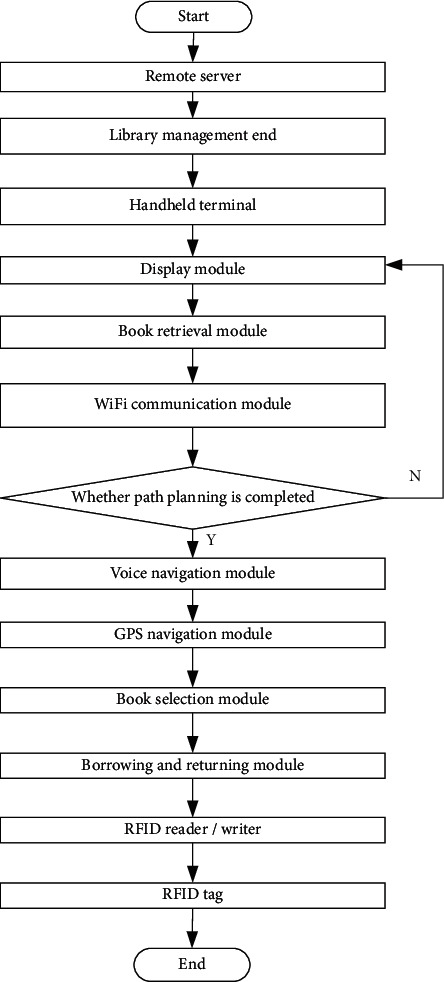
Functional component analysis flow chart of library cross-platform information retrieval module.

The functional modules of the library cross-platform information retrieval system are divided into mirror compilation sub-module, application deployment sub-module, user interaction module, application package management module, dependency management module, and mirror management module. The specific process is shown in the Figure 5.

**Figure 6:**
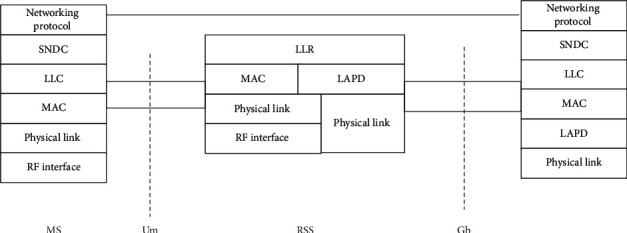
Kubernetes structure diagram of library cross-platform information retrieval system.

Among them, the mirror compilation sub-module is controlled by code version management and generates the mirror image through Dockerfile, which is also within the management scope of Kubernetes cluster. The network design of library cross-platform information retrieval platform is carried out by ZigBee and GPRS networking technologies [18]. The ZigBee data acquisition node of library cross-platform information retrieval is designed as the bottom node of library cross-platform information retrieval system, and the data of library cross-platform information retrieval is uploaded to the central server through GRPS. The original data collection, local information processing, and information fusion of library cross-platform information retrieval are realized at the sensor node [19–21]. The Kubernetes structure diagram of library cross-platform information retrieval system is shown in Figure 6.

In Figure 6, TCP/IP and X.25 protocols are adopted to realize the physical layer access and RF interface output control of the library cross-platform information retrieval system, and the distributed networking scheme is adopted to realize the online scheduling and resource virtualization configuration of the library cross-platform resources under MVB bus control protocol [22].

## 3. Mathematical Modeling of Cross-Platform Information Retrieval Model in Library

### 3.1. Feature Extraction of Retrieval Information

**Figure 7:**
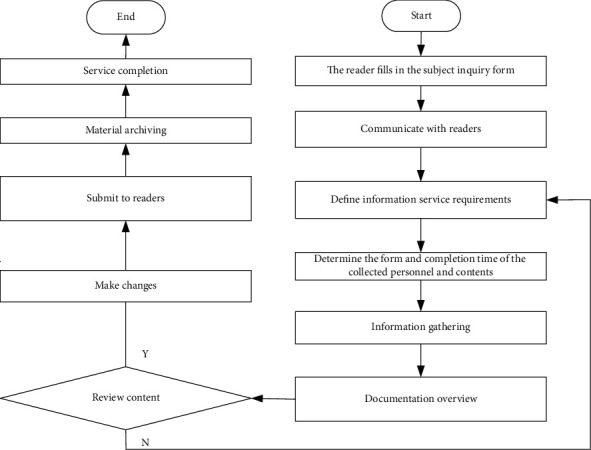
Flow chart of feature extraction for cross-platform information retrieval in library.

Based on the balanced allocation method of library cross-platform information retrieval physical space resources, the balanced allocation of library cross-platform resources is carried out [23], as shown in the Figure 7.

In the cross-platform data computing center of the library, a dynamic allocation model of resources between physical machines and virtual machines is established, and the physical machine set of the cross-platform data center of the library is *G*(*O*) = (*V*, *E*, *L*, *μ*, *η*) , *η* : *E*⟶*L*_*E*_ , and *V*, *E*, *L*, *μ*, *η* are the data set of CPU, memory, bandwidth, and hard disk resources in virtual machines, which, respectively, represent the corresponding CPU, memory, bandwidth, and hard disk resource data parameters [24]. Data set *G*_1_ = (*M*_1_^*α*^, *M*_1_^*β*^, *Y*_1_) , *a*_*n*_*i*_^*t*^_(*i* = 1,2,…, *m*) are introduced into different library cross-platform information retrieval container numbers, and the number of hard disk resources as the library cross-platform information center constitutes a feature set. At the information sampling time *t* and *t* + *τ*, the clustering center is initialized to satisfy *t*_*i*_, so that

(1) $$\begin{array}{c} {\dot{m}}_{i}\left(t\right)=-a_{i}m_{i}\left(t\right)+b_{i}\left(p_{i}\left(t-\sigma \right),p_{2}\left(t-\sigma \right),\ldots ,p_{n}\left(t-\sigma \right)\right),\\ {\dot{p}}_{i}\left(t\right)=-c_{i}p_{i}\left(t\right)+d_{i}m_{i}\left(t-\tau \right), \end{array}$$

is the fuzzy clustering center of user behavior attribute data feature vector in library cross-platform information retrieval. The attribute set of library cross-platform storage distribution space is obtained by using the feature sequence training reconstruction method of user behavior attribute data. *B* = {*b_1_, b_2_, ..., b_m_*} is the attribute category set of the cross platform information retrieval user behavior attribute data of the library to be mined. Calculate the utilization rate of the physical machine at the time of initialization or migration, and obtain that the central link distribution of the cross platform switch of the library is *a_i_*. The deployment attribute value of the library cross platform information retrieval on the virtual machine *M* is {*c_1_, c_2_, ... c_k_*}. According to the deployment of library cross-platform information retrieval nodes on virtual machine *M*, the information entropy is obtained. By using the methods of information entropy feature extraction and dissimilarity measurement, the resource scheduling fuzzy set $\left(u,u_{t}\right)\in C_{t}\left(K,{\dot{H}}_{x}^{s_{c}}\times {\dot{H}}_{x}^{s_{c}-1}\right)$ is obtained, the workload sum is obtained, and the CPU utilization rate of the moment is analyzed to obtain the control constraint parameters of library cross-platform information retrieval. The cross-platform running data of the library is divided into decision-making data, computing resource pool data, and Hadoop parallel loading data. By adopting digital twin technology and data clustering method, the digital twin parallel retrieval and information fusion feature matching model of the cross-platform information retrieval system of the library is established, and the information entropy analysis model of the cross-platform resource scheduling of the library is obtained, and the optimized analytical feature components of the cross-platform resource control of the library satisfy:

(2) $$\begin{array}{c} \left(u_{k},\Sigma_{k}\right)∼NiW\left(v_{k},V_{k}\right), \end{array}$$

where in

(3) $$\begin{array}{c} \left\{\begin{array}{c} u_{k}|\Sigma_{k}∼N\left({\hat{u}}_{k},{\hat{\Sigma }}_{k}\right),\\ \Sigma_{k}∼iW\left(v_{k}-d-1,\Lambda_{k}\right), \end{array}\right. \end{array}$$

where in *iW*(.) represents the conduction information function of library cross-platform resource scheduling, parameters *v*_*k*_ and *V*_*k*_ represent the association rule set of library cross-platform resource allocation, *d* is the information entropy dimension, ${\hat{u}}_{k}$ is the load of library cross-platform physical machines, and ${\hat{\Sigma }}_{k}$ is the dynamic load balancing parameter of library cross-platform resources. Aiming at the checkpoint of regular backup of metadata in HDFS, the library cross-platform information output rule set of each physical machine is obtained as follows:

(4) $$\begin{array}{c} p\left(x_{k}|X_{k-1},Y_{k-1}\right)=p\left(x_{k}|x_{k-1},y_{k-1}\right),\\ p\left(y_{k}|X_{k},Y_{k-1}\right)=p\left(y_{k}|x_{k}\right), \end{array}$$

where in *x*_*k*_ is the sum of the cross-platform information loads of *T* library, *y*_*k*−1_ is the utilization rate of all containers collected by the cross-platform information scheduling server of the library, and *X*_*k*−1_ and *Y*_*k*−1_ are the simulated minimum physical machine loads and energy consumption, respectively. The cross-platform running data of the library is divided into decision-making data, computing resource pool data, and Hadoop parallel loading data, and the model parameters of cross-platform information retrieval of the library are constructed based on digital twin technology estimation by adopting energy consumption simulation and sorting methods [25].

### 3.2. Cross-Platform Information Retrieval System of Library

The digital twin parallel retrieval and information fusion feature matching model of the cross-platform information retrieval system of the library is established, and the four-dimensional parameters of the cross-platform information entropy distribution of the library by the dynamic allocation of multiple complex and balanced task scheduling sequences is given, which are described as follows:

1. Random: the random migration target under the guarantee of task service quality.
2. FirstFit: the optimization fitness value of library cross-platform scheduling according to the physical machine of the data center.
3. MostFull: Calculate the initial target probability density parameters of all available physical machines, indicating the maximum information entropy under the polling scheduling mechanism.
4. LeastFull: Calculate the information entropy and waiting time of all available library cross-platform information.

Based on the construction of 4-dimensional parameters, under multiple Load Balance Service (LBS) mechanisms, the container complex balanced scheduling is carried out. Based on the digital twin technology estimation, the algorithm implementation steps of the library cross-platform information retrieval system are described as follows:

  Step 1. Put the idle and closed physical machines into the resource scheduler at the cross-platform resource control end of the library, and get the load parameter *X*.
  Step 2. The task quality mechanism is sorted by the maximum load to obtain the library cross-platform resource feature distribution set *E*, and the CPU utilization rate of each physical machine in the container *E* is estimated based on digital twin technology.
  Step 3. Move the feature quantity of library cross-platform information retrieval resources in *E* into *X*.
  Step 4. Set a proprietary conversion engine between the data source and the target data warehouse, and use the round-robin mechanism to calculate the energy consumption of the cross-platform resource retrieval of the library in *E*, so as to obtain the maximum complex resource ranking.
  Step 5. NameNode receives the notification from Secondary NameNode to generate a new log file. Select the physical machine with the smallest simulation value in *E*.

According to the above algorithm design, the library cross-platform information retrieval channel is dynamically allocated through multiple complex and balanced task scheduling sequences, and the library cross-platform information retrieval queue configuration model [26] is adopted to realize the optimal design of the library cross-platform information retrieval system.

## 4. Simulation and Result Analysis

In order to verify the application performance of this method in realizing library cross-platform resource scheduling and monitoring, the following experiments are carried out with reference [4] and reference [5] as comparative methods.

### 4.1. Establishment of Experimental Platform

First, the HBase high availability cluster is built. The regionserver of HBase is deployed on the three dat nodes of the Hadoop cluster. Each node backs up each other to ensure high availability of data. HBase's HMaster service is deployed on two NameNode nodes of HDFS, and deploying two HMasters can ensure the high availability of the cluster and prevent single-point failure. Here, an independent ZooKeeper cluster is used, but the ZooKeeper that comes with HBase is not used. When creating an HBase table, if the preallocated Region is not specified, a Region will be created by default. When massive data are written concurrently, all the data will be written into the default Region. Only when it is known that it cannot be loaded will the Split operation be performed, and it will be divided into two regions. In this process, there will be two problems: the data are all stored in one Region, which is prone to single node failure, thus affecting the whole storage operation. The underlying disk Split operation will consume a lot of cluster IO resources. Based on this problem, this paper integrates the RowKey characteristics of the cross-platform resource table of the library, designs a reasonable pre-partition scheme, solves the hot issues by creating multiple empty Region in advance, and adjusts the load balance of the cluster. Reasonable design of RowKey can make the concurrent requests in each Region evenly distributed (tend to be even), so that the IO efficiency can reach the highest. Matlab is used for simulation test. The number of cross-platform nodes in the library is 1200, the length of cross-platform information resource distribution sampling in the library is 1024, the number of training samples is 200, the data size is 800 Mbyte, and the maximum memory buffer capacity of each library is 5600. See Table 1 for the task allocation parameters of cross-platform resource scheduling in the library.

### 4.2. Cross-Platform Resource Allocation Histogram of Library

**Figure 8:**
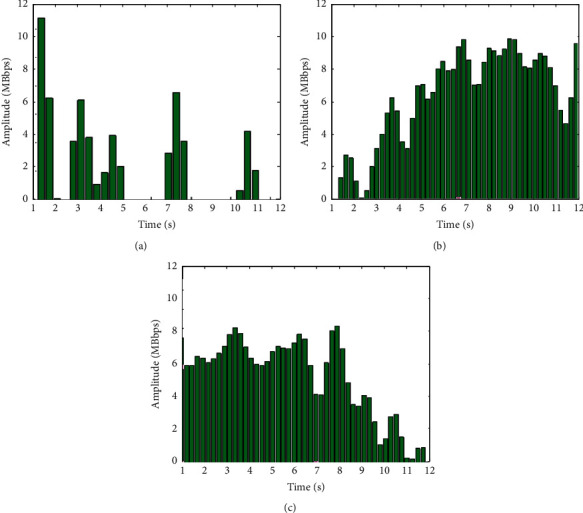
Histogram sequence of cross-platform resource allocation in library. (a) Platform1. (b) Platform2. (c) Platform3.

According to the parameter configuration in Table 1, the library cross-platform resources are monitored, and the histogram sequence of library cross-platform resources configuration is shown in Figure 8.

### 4.3. Experimental Results and Analysis

**Figure 9:**
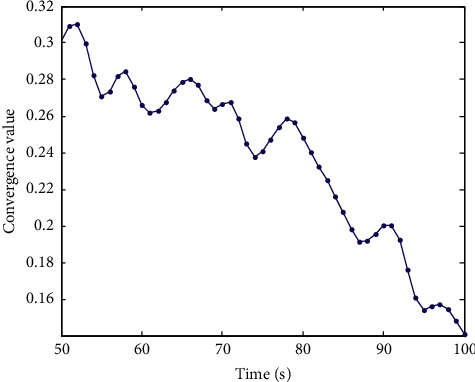
Convergence curve of resource allocation.

According to the library cross-platform resource allocation in Figure 8, the library cross-platform information retrieval queue configuration model is adopted to realize the optimal configuration of the library cross-platform information retrieval system. Taking the convergence value as the index, the resource allocation of this method is tested after 100 s. The faster the convergence speed is, the better the resource allocation performance of this method is. The convergence curve of resource allocation is shown in Figure 9.

By analyzing Figure 9, it can be concluded that the cross-platform information retrieval of the library by this method has good convergence for resource allocation.

**Figure 10:**
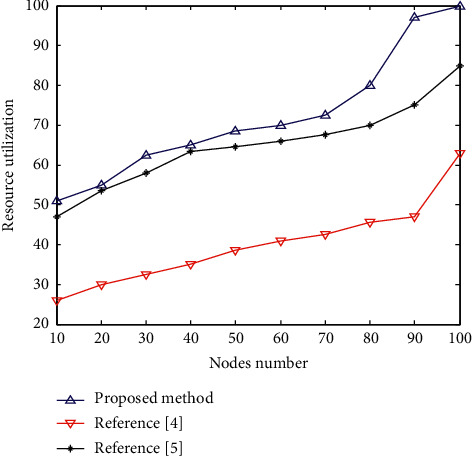
Comparative test of resource utilization rate.

After the application of this method [4] and [5], the library information retrieval resource utilization rate of 100 nodes is tested. The higher the value, the higher the resource utilization rate of the corresponding method and the better the retrieval performance. The comparison results are shown in Figure 10.

Analysis of the simulation results in Figure 10 shows that this method has better data recall ability for library cross-platform information retrieval, which improves the resource utilization rate by 32.3% compared with the traditional method.

## 5. Conclusions

In order to improve the performance of cross-platform library information retrieval, this paper designs a library cross-platform information retrieval system based on digital twins. The following conclusions can be drawn from the above research:

1. This paper constructs an optimized library cross-platform information retrieval and queue scheduling model, and controls and stores the library cross-platform information resource allocation through network server transmission.
2. The test shows that the resource utilization rate of information retrieval is higher and the convergence of resource allocation is better in 10 library cross platforms.
3. The next step is to refine the library information to further improve the retrieval performance of the design system.

## Figures and Tables

**Table 1:** Task allocation parameters of library cross-platform resource scheduling.

Platform	Memory buffer capacity/mb	Retrieve task queue length/kbps
Platform1	16.840	799.891
Platform2	868.511	472.017
Platform3	556.266	461.907
Platform4	495.678	277.859
Platform5	717.503	169.823
Platform6	99.289	65.178
Platform7	804.483	87.130
Platform8	959.692	803.154
Platform9	984.799	898.029
Platform10	801.790	151.836

## Data Availability

The raw data supporting the conclusions of this article can be obtained from the corresponding author without undue reservation.
